# Temperature- and Size-Dependent Photoluminescence of CuInS_2_ Quantum Dots

**DOI:** 10.3390/nano13212892

**Published:** 2023-11-01

**Authors:** Oleg Korepanov, Dmitriy Kozodaev, Olga Aleksandrova, Alexander Bugrov, Dmitrii Firsov, Demid Kirilenko, Dmitriy Mazing, Vyacheslav Moshnikov, Zamir Shomakhov

**Affiliations:** 1 Micro- and Nanoelectronics Department, Saint Petersburg Electrotechnical University “LETI”, 197022 Saint Petersburg, Russia; kozodaev@ntmdt.nl (D.K.); oaaleksandrova@gmail.com (O.A.); d.d.firsov@gmail.com (D.F.); dmazing@yandex.ru (D.M.); vamoshnikov@mail.ru (V.M.); 2 NT-MDT BV, 7335 Apeldoorn, The Netherlands; 3 Department of Physical Chemistry, Saint Petersburg Electrotechnical University “LETI”, 197022 Saint Petersburg, Russia; anbugrov@etu.ru; 4 Ioffe Institute, 194021 Saint Petersburg, Russia; zumsisai@gmail.com; 5 Institute of Informatics, Electronics and Robotics, Kabardino-Balkarian State University, n.a. Kh.M. Berbekov, 360004 Nalchik, Russia; shozamir@yandex.ru

**Keywords:** quantum dots, CuInS_2_, temperature-dependent photoluminescence

## Abstract

We present the results of a temperature-dependent photoluminescence (PL) spectroscopy study on CuInS_2_ quantum dots (QDs). In order to elucidate the influence of QD size on PL temperature dependence, size-selective precipitation was used to obtain several nanoparticle fractions. Additionally, the nanoparticles’ morphology and chemical composition were studied using transmission electron microscopy, X-ray diffraction, and X-ray photoelectron spectroscopy. The obtained QDs showed luminescence in the visible–near infrared range. The PL energy, linewidth, and intensity were studied within an 11–300 K interval. For all fractions, a temperature decrease led to a shift in the emission maximum to higher energies and pronounced growth of the PL intensity down to 75–100 K. It was found that for large particle fractions, the PL intensity started to decrease, with temperature decreasing below 75 K, while the PL intensity of small nanoparticles remained stable.

## 1. Introduction

Quantum dots (QDs) are nanocrystals composed of semiconductor materials that exhibit photoluminescent properties, making them increasingly popular in research and development. Their unique optoelectronic properties make them highly attractive for numerous applications. QDs come in various sizes, shapes, and chemical compositions, and can be employed in a number of different spheres, from solid-state lighting and photovoltaics to life science applications, such as imaging, sensing, medical diagnostics, and photodynamic therapy. With current research focusing on the use of nanocrystals with dimensions of less than 10 nm, these materials offer size-dependent absorption and emission energy tunability that is beneficial for development of bio-labeling reagents, as well as photonic devices such as liquid-crystal displays, QD-solar cells, light-emitting diodes (LEDs), and photodetectors [1,2,3,4,5].

QDs composed of group II–VI elements, e.g., cadmium and lead chalcogenides, have become the most popular choice due to their tunable absorption onset and strong fluorescence. However, the obvious toxicity of these materials has limited their use in some areas [6,7]. Thus, binary compound semiconductors (e.g., InP and ZnS) [8], elemental semiconductors (e.g., Si, C) [9], and ternary chalcogenides [10], which offer comparable optical features and tunable emission and absorption wavelengths, without the same level of toxicity, have been explored. The search for safer and earth-abundant QD materials is a current research topic. This includes ternary nanocrystals such as the CuInS(Se)_2_ and AgInS(Se)_2_, as well as the quaternary nanocrystals Zn-Cu-In-S(Se) and Zn-Ag-In-S(Se).

CuInS_2_ is a ternary I–III–VI semiconductor with direct transition bandgap energy of 1.53 eV. CuInS_2_ and AgInS_2_ nanocrystals have broad photoluminescence (PL) spectra with a full width at half maximum (FWHM) of around 0.4 eV. Their PL quantum yields (PLQY) can be enhanced through composition control, while their absorption and emission spectra show a blue shift with decreasing particle size [11]. Recent advancements have demonstrated potential for ternary and quaternary nanocrystals based on I–III–VI semiconductors in novel applications such as photovoltaics and photocatalysis, as well as biomedicine (e.g., multimodal imaging, drug delivery, theranostics, etc.) [12,13,14].

While the most common approach to QD preparation is synthesis in organic solvents, yielding hydrophobic QDs with nonpolar surface ligands, ternary and quaternary QDs can be synthesized in water. Rendering QDs synthesized in nonpolar medium dispersible in water could be performed via a ligand exchange process; however, this can have detrimental effects on the photophysical properties of nanocrystals, inducing the formation of new trap states that promote the nonradiative recombination of charge carriers, and thus, leading to photoluminescence quenching. Aqueous techniques become even more relevant in the case of I–III–VI nanocrystals, which are typically synthesized in nonpolar media employing aliphatic thiols as coordinating ligands, resulting in the strong coordination of surface groups and hindering ligand exchange. The synthesis of ternary and quaternary QDs in aqueous medium can be achieved using a various types of capping ligands such as thiols [15,16], amino-acids [17,18,19], polymers [20,21], etc., providing excellent long-term colloidal stability.

Luminescence thermometry is a flexible optical method that measures local temperatures, where temperature-dependent changes function as indicators or probes. Rare-earth ion-doped luminescent compounds have become a popular alternative for making temperature-dependent sensors [22,23]. The single emission center that the phosphor-based optical temperature detector relies on makes it unstable and inaccurate due to variations in excitation power [24]. Compared to rare-earth ion-doped phosphors, QDs demonstrate high sensitivity and photostability, although the potential application of QDs in optical temperature sensing, as well as the underlying mechanisms, require further investigation.

Several studies discuss the impact of particle size and low temperatures on the optical characteristics of QDs [25,26,27]. However, the correlation between particle size and temperature dependence remains inconclusive. In this study, CuInS_2_ QDs were synthesized and separated according to size, and temperature curves were obtained within the range of room temperature to 11 K.

## 2. Materials and Methods

### 2.1. Chemicals

Indium(III) chloride (InCl_3_, 99.999%) and 3-Mercaptopropionic acid (C_3_H_6_O_2_S, 99%) were purchased from Sigma Aldrich (St. Louis, MO, USA). Copper(II) acetate (Cu(CH_2_COO)_2_, 99%), sodium sulfide (Na_2_S∙9H_2_O, 98%), and sodium hydroxide (NaOH) were provided by Vekton (St. Petersburg, Russia). All chemicals were used without additional purification.

### 2.2. Synthesis of CuInS_2_ QDs

In the presented work, MPA was used as a stabilizer. A distinctive feature of MPA is its relatively small molecule size compared to known surfactants used in the synthesis of I–III–VI QDs (L-Glutathione, polymers, etc.). The use of short-chain molecules makes it possible to reduce the distance between QDs, which leads to an increase in charge transfer efficiency.

The photoluminescent quantum yield and photostability of ternary QDs can be adjusted through composition variation. Generally, the highest PL quantum yield and photostability observed in I–III–VI QDs are achieved at a molar ratio of [I]:[III] = 1:4 [28,29].

CuInS_2_ QDs were synthesized in an aqueous solution. Under magnetic stirring, 0.1 mmol of Cu(CH_2_COO)_2_, 0.4 mmol of InCl_3_, and 2 mmol of MPA were dissolved in 5 mL of deionized water. The pH of the solution was adjusted to 7.00 using 0.1 M NaOH; then, a freshly prepared aqueous solution containing 0.5 mmol of Na_2_S·9H_2_O was added. The solution was heated to 95 °C for 300 min.

QDs of different sizes were obtained via n steps of size-selective precipitation, where n represents the fraction number. Precipitation was achieved through the addition of isopropanol, and separation was performed via centrifugation.

### 2.3. Methods

Transmission electron microscopy (TEM) studies were performed using a JEOL JEM-2100F microscope, JEOL, Akishima, Japan (accelerating voltage 200 kV, point resolution 0.19 nm). Specimens for the TEM were prepared via wetting of a conventional copper TEM grid covered with a carbon lacey film in suspension and its subsequent drying in air.

X-ray diffraction measurements of the samples were carried out using a Rigaku SmartLab diffractometer (Rigaku Corporation, Tokyo, Japan) in a 2θ angle range of 15 to 60° with a scanning rate of 1° per minute using CuK_α_ radiation at 40.0 kV and 30.0 mA. Qualitative X-ray phase analysis of the QDs was performed by comparing the experimental diffraction patterns with the cards of the International Centre for Diffraction Data’s PDF databases using the Crystallographica Search-Match program. The average size of the coherent scattering regions was calculated through the broadening of X-ray diffraction lines using the Rigaku SmartLab Studio II software package version 2.0.

X-ray photoelectron spectroscopy (XPS) spectra were recorded using a “K-Alpha” Thermo Scientific system (Waltham, MA, USA) equipped with an Al-K_α_ (1486.6 eV) X-ray source. The sample was outgassed to less than 4.5 × 10^−9^ mbar. The background subtraction of secondary electrons was performed using the Shirley method. The spectrometer energy scale was calibrated with Au 4f_7/2_, Cu 2p_3/2_, and Ag 3d_5/2_ lines at 84.2, 367.9, and 932.4 eV, respectively.

The infrared transmission measurements were performed using a Vertex 80 Fourier transform infrared (FTIR) spectrometer (Bruker Optics, Ettlingen, Germany) equipped with a SiC globar as the IR radiation source, a KBr beamsplitter, and a pyroelectric deuterated L-alanine-doped triglycine sulphate (DLaTGS) photodetector. A PL measurement setup [30] based on the same FTIR spectrometer (equipped instead with a CaF_2_ beamsplitter and a Si diode photodetector) was employed to obtain the temperature dependences of the PL spectra. The samples were placed into a Janis CCS-150 closed-cycle helium cryostat, and a 405 nm semiconductor laser diode was used as an excitation source. Nanoparticle samples were dried, powdered, and made into transparent films by mixing them with KBr. The QD solutions were poured into quartz cuvettes and measurements were performed using the same PL spectra measurement setup without cryostat implementation.

Absorption spectra of QD solutions were acquired using a PE-5400UV UV-vis spectrophotometer (“Ekohim” LLC, St. Petersburg, Russia) in quartz cuvettes.

## 3. Results and Discussion

### 3.1. Structure Characterization

In order to establish the morphology and structure of the CuInS_2_ QDs, TEM and XRD studies were performed.

**Figure 1:**
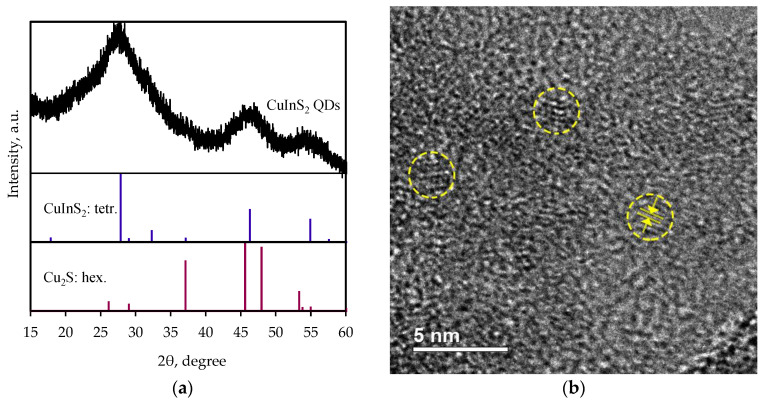
(**a**) XRD patterns and (**b**) TEM image of obtained samples. In (**b**), several quantum dots are encircled in yellow, while the interplanar spacing is indicated by the arrows.

The X-ray diffraction pattern (Figure 1a) contains at least three broad peaks in the 2θ angle ranges of 23–34°, 42–50°, and 52–58°, which indicates the presence of a crystalline structure in the sample. The observed Bragg peaks are in good agreement with the (112), (200), (204), (220), and (312) planes of CuInS_2_ (JCPDS 65-2732) with the chalcopyrite structure, which indicates that the resulting QDs are nuclei of the tetragonal crystalline phase CuInS_2_ (Figure 1a). In this case, characteristic reflections of the hexagonal Cu_2_S phase (JCPDS 84-0208) are not observed in the sample. The average size of the coherent scattering regions (D) of CuInS_2_, calculated using the Rigaku SmartLab Studio II program through the broadening of X-ray maxima, is approximately 1.5 nm. The average QD size of 2 nm, estimated via XRD, is in an agreement with the TEM results. According to TEM results (Figure 1b), chalcopyrite planes corresponding to (112) with a period of 0.32 nm are observed, and the QDs have an average diameter of 2 ± 0.5 nm. The average size of the obtained QDs is less than the Bohr radius of exciton in CuInS_2_, which means that the nanoparticles are in a strong confinement regime.

**Figure 2:**
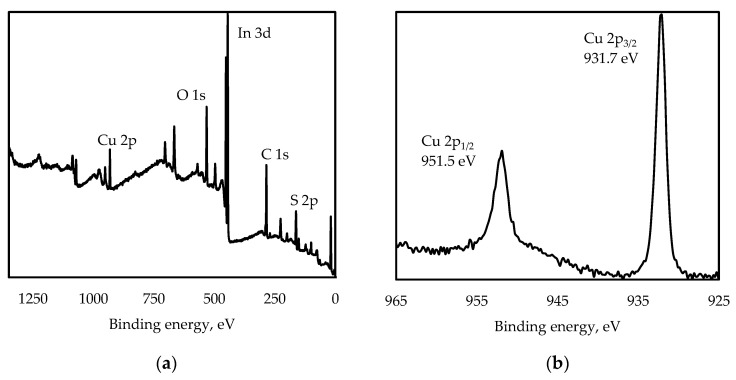

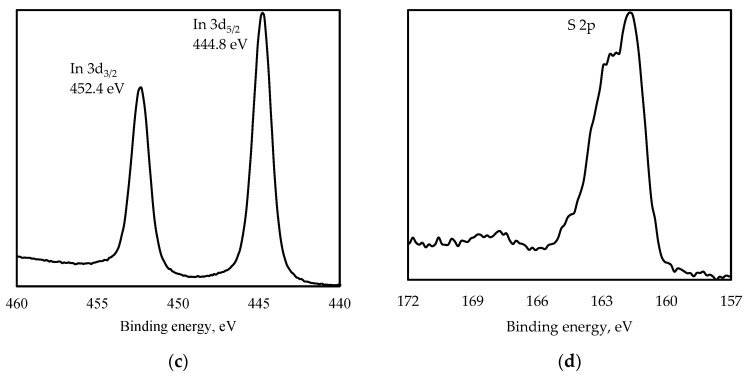
XPS spectra of CuInS_2_ QDs: survey (**a**), Cu 2p (**b**), In 3d (**c**), S 2p (**d**).

Figure 2 shows the XPS spectra of the CuInS_2_ QDs.

**Figure 3:**
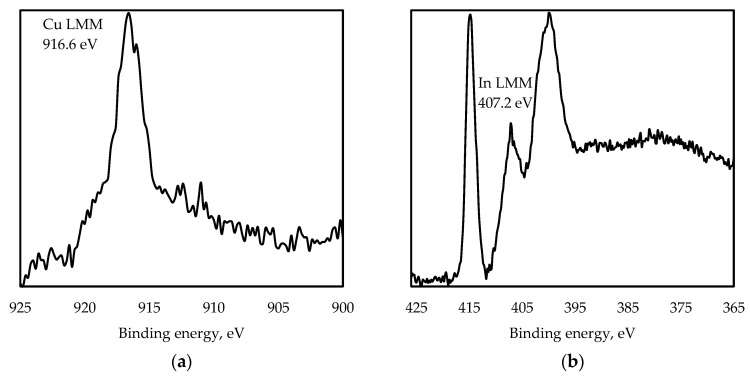
XPS signal corresponding to Cu LMM (**a**) and In LMM (**b**) Auger transitions of CuInS_2_ QDs.

From Figure 2, it can be seen that Cu 2p peaks are found at 931.7 eV (Cu 2p_3/2_) and 951.5 eV (Cu 2p_1/2_), In 3d peaks are found at 444.8 eV (In 3d_5/2_) and 452.4 eV (In 3d_3/2_), and an S 2p peak is found at 161.7 eV. C and O elements comprising MPA ligands are detected, as well (Figure 2a). In Figure 2b, the Cu 2p line is split into Cu 2p_3/2_ and Cu 2p_1/2_ (splitting value is 19.8 eV), with no distinguishable Cu^2+^ peak at about 942 eV. In Figure 3a, the kinetic energy of the Cu LMM Auger transition (916.6 eV), corresponding to Cu_2_O, suggests that the copper is in the Cu^+^ state in this sample. The In 3d line is split into In 3d_5/2_ and In 3d_3/2_, with splitting energy of 7.6 eV (Figure 3b). Two peaks, In 3d_5/2_ and In 3d_3/2_, and the kinetic energy of the In M_4_N_45_N_45_ Auger transition of 407.2 eV (Figure 3b) are assigned to In^3+^ in the CuInS_2_ QDs. Two distinct peaks at binding energies of 161.7 and 162.8 eV are assigned to S 2p_3/2_ and S 2p_1/2_, which is consistent with the values for S^2−^ [31].

### 3.2. Absorption and PL Spectra

**Figure 4:**
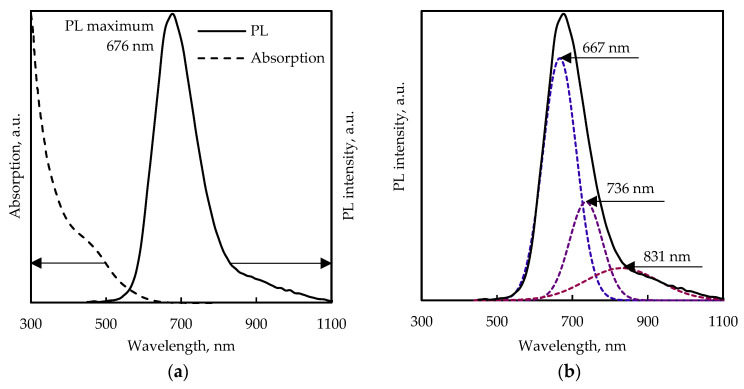
PL (solid line) and absorption spectra (dash line) of the CuInS_2_ QD stock solution (**a**) and PL spectrum deconvolution result (**b**).

Figure 4 shows the PL spectrum of the obtained CuInS_2_ QD aqueous solution at room temperature and its deconvolution result, as well as the absorption spectrum of the same solution.

The obtained sample shows features common to I-III-VI QDs, such as the absence of an absorption exciton peak and a broad PL band with a large Stokes shift. The PL curve, represented by a peak with a maximum at about 676 nm and an FWHM parameter at about 115 nm, can be qualitatively decomposed as the sum of three Gaussian functions with maxima at about 667, 736, and 831 nm. It can be assumed that the radiative recombination in the studied sample is due to three mechanisms. The large Stokes shift, which is about 290 meV, indicates the emission mechanism associated with defects, and the absence of the interband radiative recombination component. There are several main theories under consideration for the observed characteristic of PL in Cu-In-S nanocrystals. In the first model, emission is associated with the trapping of photoexcited charge carriers on donor and acceptor defects, followed by radiative recombination (a mechanism involving donor–acceptor pairs) [32]. The transition energy in this case also depends on the Coulomb interaction of ionized defects, and hence, on their relative spatial localization, which is a source of additional broadening of the PL spectrum. The acceptor levels can be represented by point defects (e.g., V_Cu_) and even group I atom centers in their regular lattice positions (Cu^+^, Cu^2+^), and the donor levels of In_Cu_, V_S_, In_i_. According to this approach, the two emission channels can correspond to the donor–acceptor pairs V_Cu_–In_Cu_ (relatively short wavelength band) and V_Cu_–V_S_ (long wavelength band). Another approach to explaining the optical properties of Cu-In-S nanocrystals involves photoexcited hole trapping at Cu^+^ sites followed by recombination with a delocalized photoexcited electron [33]. In this case, the Stokes shift is governed by the strong electron–phonon interaction, and the dependence of the transition energy on the crystal size can be easily explained by the change in the energy of the LUMO orbital due to quantum confinement. This model is in good agreement with the results of cyclic voltammetry studies and time-resolved absorption spectroscopy [33,34]. It cannot be ruled out that both mechanisms coexist simultaneously. States associated with surface defects may also be involved in the process of radiative recombination.

**Figure 5:**
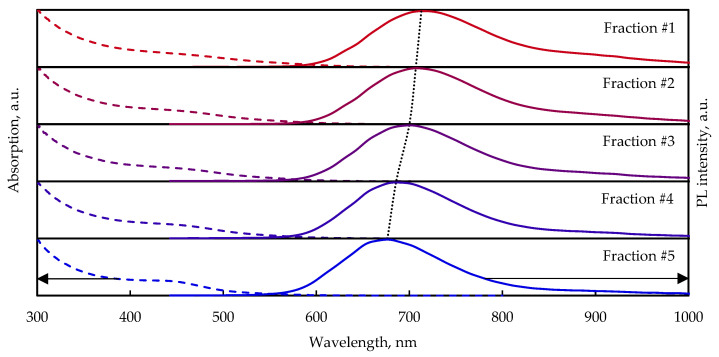
PL (solid lines) and absorption spectra (dash lines) of size-selected CuInS_2_ QDs in aqueous solution.

The PL spectra of the size-dependent CuInS_2_ QDs are shown in Figure 5.

Each consecutive precipitation yields a smaller average size fraction (designated fraction ordered from the largest (#1) to the smallest (#5), leading to a blue shift in the PL spectrum (from 713 nm to 677 nm), which, assuming that PL occurs via recombination of the localized hole with the delocalized electron, is caused by a rise in the conduction band bottom due to the effect of spatial quantization [35]. There is a tail extending from 850 nm on the PL spectra of every sample caused by emission through the surface states. According to the TEM images (Figure S1), the particles in fraction #1 are noticeably larger, with sizes from around 4 nm; in fraction #5, the particles do not exceed 3 nm.

### 3.3. Temperature-Dependent Photoluminescence

**Figure 6:**
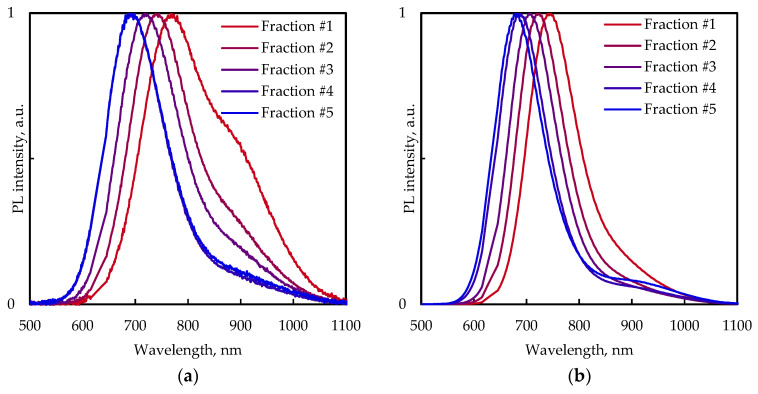
Normalized PL spectra of size-selected CuInS_2_ QDs in KBr pellets at 300 K (**a**) and 11 K (**b**).

Nanoparticle samples were dried, powdered, and made into transparent films by mixing them with KBr. Figure 6 shows the PL spectra of the CuInS_2_ QDs of separated fractions at room temperature and 11 K.

The PL spectra of size-selected CuInS_2_ QDs in KBr pellets at 300 K have at least two PL bands in range of 690–770 nm and around 900 nm. For fractions #1 and #2 (large particles) at room temperature, the shoulder at around 900 nm is more pronounced than for fractions #3–#5. In contrast, the shoulder becomes more distinct with decreasing temperature for fractions #4 and #5.

**Figure 7:**
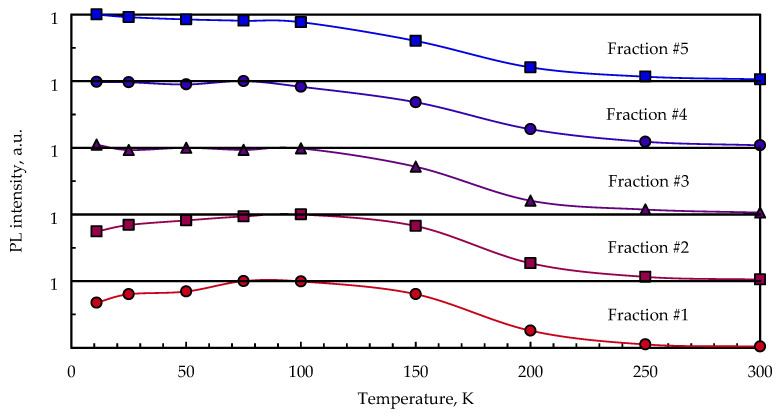
Temperature dependence of the PL integral intensity of size-selected CuInS_2_ QDs in KBr pellets.

The general trend of the blue shift of PL spectra with decreasing QD size is retained for the samples prepared in a KBr pellet. The PL peak position shifts from around 1.61 eV for fraction #1 to 1.78 eV for fraction #5 at room temperature (Figure 6a) and from 1.66 eV to 1.82 eV at 11 K (Figure 6b). The PL spectra curves of fractions #4 and #5 of the CuInS_2_ QDs in the KBr pellet almost coincide at room temperature. Upon cooling down to 11 K the PL maximum positions of these fractions slightly diverge and the PL spectra of these fractions are well resolved (Figure 7).

The profile of the PL spectra of the CuInS_2_ QD samples exhibits strong temperature dependence in the range of 11–300 K. The temperature decrease leads to a shift in the emission maximum to higher energies and pronounced growth of the PL intensity up to 75–100 K. A further decrease in PL intensity with decreasing temperature occurs for fractions #1 and #2 (large particles), while no such trend is observed for fractions #3, #4, and #5 (small particles), which does not experience PL intensity change below 75 K.

The temperature dependence of the integral PL intensity could be associated with the recombination rate (Equation (1)). The thermal dependence of the recombination rate has an activation characteristic and includes the radiative and nonradiative parts (Equation (2)). As the nonradiative component of the decay rate goes down with decreasing temperature, the intensity dependence reaches saturation below 100 K (becoming solely a function of the radiative decay rate, which is temperature-independent).

(1) $$I\left(t\right)=\sum_{i=1}^{3}a_{i}e^{\left(-\frac{t}{\tau_{i}}\right)},$$

(2) $$\frac{1}{\tau \left(T\right)}=\frac{1}{\tau_{r}}+\frac{1}{\tau_{\mathrm{nr}}\left(0\right)}e^{\left(-\frac{E_{a}}{k_{B}T}\right)},$$

In the above equations, τ_r_ and τ_nr_ are the radiative and nonradiative decay times, respectively, E_a_ is the thermal activation energy, and k_B_ is the Boltzmann constant.

The observed decrease in PL intensity below 100 K in larger fractions of QDs of the I–III–VI ternary systems may be explained in view of the assumption that the radiative decay time increases with increasing nanocrystal size due to an increasing ligand-to-QD ratio. In our case, molecules up to 700 MPA can coordinate QDs from a small fraction, and molecules up to 1200 MPA can bind to QDs from a large fraction. An increased ligand-to-QD ratio resulted in a higher probability of the charge carrier trapping process occurring, which, for small QDs, leads to decreasing PL lifetimes.

Assuming the DAP emission model, the radiative recombination rate (1/τ_r_) should depend on the distance between the donor and acceptor centers. Given that there are numerous emission channels with different rates, the PL decay profile is multiexponential. In [36], the authors analyzed the temperature dependence of the PL of silicon nanoparticles of different sizes in a temperature range of 5–300 K. The authors also observed a decrease in PL intensity with decreasing temperature below 70 K. A less pronounced decrease in PL intensity was registered for small nanoparticles. The authors also provided time-resolved PL measurements for the samples, and found a correlation between particle size and PL quenching rate. At a certain temperature, the large crystals experienced a greater saturation effect than the smaller ones due to their longer PL lifetime. In CuInS_2_ QDs, the decay time of PL similarly decreased with decreasing particle size [37]. Accordingly, these considerations could be applied to explain the temperature dependence of integral PL intensity in our samples.

**Figure 8:**
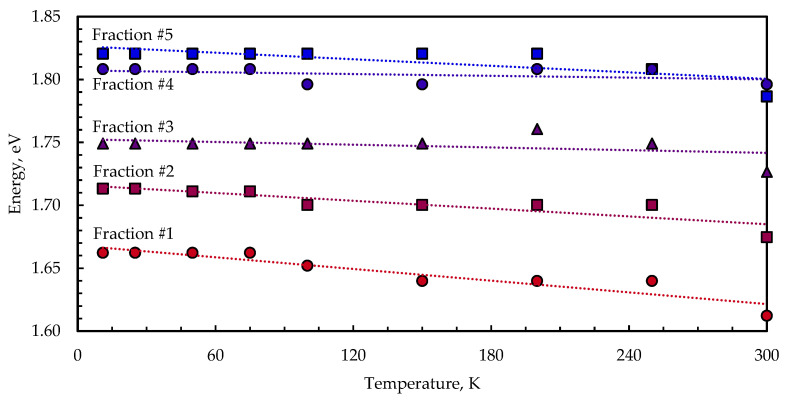
Temperature dependence of the PL peak maximum of size-selected CuInS_2_ QDs in KBr pellets.

Figure 8 shows the temperature-dependent PL emission maximum shift for five size-selected samples of CuInS_2_ QDs embedded in KBr. As the temperature increases, all the samples show a decrease in emission energy of several tens of meV. A similar trend was observed in nanocrystalline binary systems [38,39,40] and self-assembled QDs [41,42], as well. This effect could be attributed to thermally induced population redistribution across inhomogeneously broadened states.

The energy of the PL peak intensity of the transitions E_max_ is determined via the convolution of the density of states (DOS) and the state population functions. The temperature dependence of E_max_ is given by the following expression:

(3) $$E_{\max}\left(T\right)=E_{0}\left(0\right)-\frac{\alpha T^{2}}{\beta +T}-\frac{\sigma^{2}}{\left(\mathrm{kT}\right)},$$

where E_0_(0) is the DOS maximum energy at 0 K, α and β are the Varshni parameters, σ is the dispersion of the DOS function, and k is the Boltzmann constant. According to (3), if an ensemble of states is broadened with the dispersion σ, the peak energy E_max_ is shifted by −σ^2^/kT relative to E_0_.

The direct band-to-band transition is not a determinant of the PL properties, and CuInS_2_ QD emission occurs due to the involvement of copper-related levels [34]. The optical properties of CuInS_2_ QDs thus strongly resemble those of copper-doped binary II–VI and III–V QDs [43]. The copper centers form optically active levels within the bandgap near the valence band edge in binary II–VI systems, e.g., ZnS, ZnSe CdS, and CdSe [44,45,46,47]. Notably, according to [44], in ZnS:Cu^2+^, copper levels shift with the valence band as the temperature changes, which allows us to assume that Varshni’s law, with some modification, is applicable to the temperature-dependent PL characterization of CuInS_2_ QDs.

**Figure 9:**
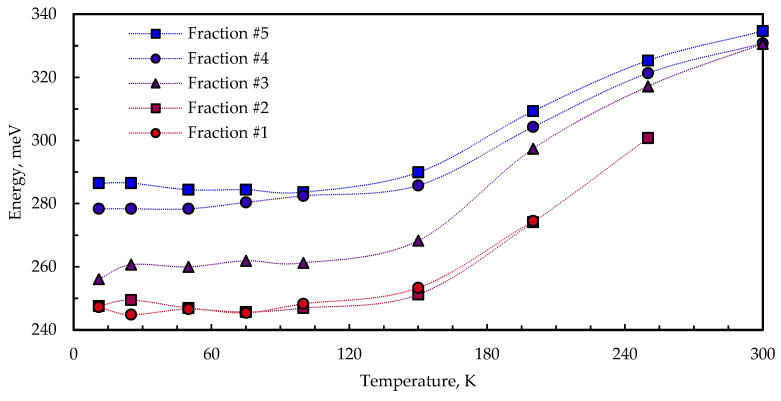
Temperature dependence of PL FWHM of size-selected CuInS_2_ QDs in KBr pellets.

Figure 9 shows the PL FWHM temperature dependence of CuInS_2_ QDs in KBr pellets for five different QD sizes. In a lower temperature range below 120 K, the dependence of FWHM on temperature is weak. A further temperature increase leads to PL curve broadening for all fractions, so that the difference between FWHMs becomes negligible at room temperature. Emission FWHM analysis with temperature variation provides information about the exciton–phonon interaction in QDs. The experimental data could be modeled using Equation (4), defining the temperature dependence of exciton peak broadening in bulk semiconductors. The total emission width is described as the sum of an inhomogeneous broadening term, which is connected with QD size distribution and terms representing homogeneous broadening, mainly due to optical phonon–exciton interactions.

(4) $$\Gamma \left(T\right)=\Gamma_{\mathrm{inh}}+\Gamma_{\mathrm{LO}}{\left(e^{\frac{E_{\mathrm{LO}}}{\mathrm{kT}}}-1\right)}^{-1}$$

$\Gamma_{inh}$—the temperature-independent intrinsic inhomogeneous line width;
$\Gamma_{LO}$—the longitudinal optical (LO) phonon–exciton coupling coefficient;
$E_{LO}$—the LO phonon energy.

Applying a numerical value of E_LO_ equal to 36 meV obtained via Raman spectroscopy in [48,49], the authors of [27] determined the values of Γ_inh_ and Γ_LO_ to be 320 meV and 0.1 meV for the whole CuInS_2_ QD stock ensemble. In our case, the values of Γ_inh_ and Γ_LO_ varied with fraction size in the ranges 245–280 meV and 80–110 meV, respectively. The assessed Γ_LO_ values appear to be too high. The broadening of the PL curve with increasing temperature is likely more complex than being attributed to electron–phonon coupling, potentially necessitating the consideration of various thermally activated emission centers. The narrowing of the PL curve with decreasing temperature may be linked to the influence of surface ligands.

In [50], the authors suggested that thiol groups could influence the PL emission properties of aqueous I–III–VI QDs through the transfer of a hole to the redox level of surface-bound disulfides formed via ligand oxidation. In our previous work [21], though, we observed very little narrowing of the PL curve with decreasing temperature for non-thiol (thiol-less) PVP-capped AgInS_2_ QDs. It is likely that the stabilization mechanism of these QDs is based on the interaction of oxygen from PVP with the QD surface. Given the higher electronegativity of oxygen, its electron-donating ability is weaker, thus making ligand oxidation (hole trapping) less probable. For the smallest QDs (fraction #5), in contrast to other fractions, the FWHM slightly increased below 100 K, which could be due to the exciton leakage effect [51].

## 4. Conclusions

In conclusion, CuInS_2_ quantum dots (QDs) were synthesized in aqueous solution utilizing MPA as a ligand. For our studies, nanoparticles of different sizes were obtained via size-selective precipitation. The temperature-dependent photoluminescence (PL) spectra of CuInS_2_ QDs were examined in the temperature range of 11–300 K. It was found that the temperature decrease led to a shift in the emission maximum to higher energies and a decrease in PL intensity down to 75–100 K, with a further decrease observed for larger nanoparticles below 75 K, whereas small nanoparticles did not display any alteration in PL intensity at lower temperatures. The shift in the PL peak towards higher energies with decreasing temperature was linked to the change in the convolution of the density of states and the state population functions. The observed PL line broadening with increasing temperature was associated with the influence of surface thiol ligands. It was discovered that the intricate relationship between integral intensity and temperature involves the size of CuInS_2_ QDs. A temperature sensor design based on QDs should consider both the dimensions and size distribution of the QDs employed. Our observations could be useful for better understanding the luminescence mechanism in CuInS_2_ QDs.

## Data Availability

The data that support the findings of this study are available from the corresponding author upon reasonable request.

## Supplementary Materials

The following supporting information can be downloaded at: https://www.mdpi.com/article/10.3390/nano13212892/s1, Figure S1: TEM image of the fraction #1 (a) and fraction #5 (b) of CuInS_2_ QDs; Table S1: PL parameters of size-selected QDs in solution; Table S2: Γ_inh_ and Γ_LO_ values calculated using Formula (4) for the different fractions; Figure S2: Normalized PL spectra of size-selected CuInS_2_ QDs in KBr pellets in temperature range of 11–300 K. Fractions #1–#5 presented in (a–e). Temperature dependence of the PL integral intensity of the same samples (f).

## References

[1] Yin X., Zhang C., Guo Y., Yang Y., Xing Y., Que W. (2021). PbS QD-Based Photodetectors: Future-Oriented near-Infrared Detection Technology. J. Mater. Chem. C.

[2] Matea C., Mocan T., Tabaran F., Pop T., Mosteanu O., Puia C., Iancu C., Mocan L. (2017). Quantum Dots in Imaging, Drug Delivery and Sensor Applications. IJN.

[3] Reshma V.G., Mohanan P.V. (2019). Quantum Dots: Applications and Safety Consequences. J. Lumin..

[4] Bera D., Qian L., Tseng T.-K., Holloway P.H. (2010). Quantum Dots and Their Multimodal Applications: A Review. Materials.

[5] Liu Z., Lin C.-H., Hyun B.-R., Sher C.-W., Lv Z., Luo B., Jiang F., Wu T., Ho C.-H., Kuo H.-C. (2020). Micro-Light-Emitting Diodes with Quantum Dots in Display Technology. Light Sci. Appl..

[6] Kauffer F.-A., Merlin C., Balan L., Schneider R. (2014). Incidence of the Core Composition on the Stability, the ROS Production and the Toxicity of CdSe Quantum Dots. J. Hazard. Mater..

[7] Zhang Y., Chen W., Zhang J., Liu J., Chen G., Pope C. (2007). In Vitro and In Vivo Toxicity of CdTe Nanoparticles. J. Nanosci. Nanotechnol..

[8] Tarantini A., Wegner K.D., Dussert F., Sarret G., Beal D., Mattera L., Lincheneau C., Proux O., Truffier-Boutry D., Moriscot C. (2019). Physicochemical Alterations and Toxicity of InP Alloyed Quantum Dots Aged in Environmental Conditions: A Safer by Design Evaluation. NanoImpact.

[9] Liu N., Tang M. (2020). Toxicity of Different Types of Quantum Dots to Mammalian Cells in Vitro: An Update Review. J. Hazard. Mater..

[10] Girma W.M., Fahmi M.Z., Permadi A., Abate M.A., Chang J.-Y. (2017). Synthetic Strategies and Biomedical Applications of I–III–VI Ternary Quantum Dots. J. Mater. Chem. B.

[11] Zhong H., Bai Z., Zou B. (2012). Tuning the Luminescence Properties of Colloidal I–III–VI Semiconductor Nanocrystals for Optoelectronics and Biotechnology Applications. J. Phys. Chem. Lett..

[12] Huang W.-T., Yoon S.-Y., Wu B.-H., Lu K.-M., Lin C.-M., Yang H., Liu R.-S. (2020). Ultra-Broadband near-Infrared Emission CuInS_2_/ZnS Quantum Dots with High Power Efficiency and Stability for the Theranostic Applications of Mini Light-Emitting Diodes. Chem. Commun..

[13] Chetty S.S., Praneetha S., Vadivel Murugan A., Govarthanan K., Verma R.S. (2020). Human Umbilical Cord Wharton’s Jelly-Derived Mesenchymal Stem Cells Labeled with Mn^2+^ and Gd^3+^ Co-Doped CuInS_2_–ZnS Nanocrystals for Multimodality Imaging in a Tumor Mice Model. ACS Appl. Mater. Interfaces.

[14] Gao X., Liu Z., Lin Z., Su X. (2014). CuInS2 Quantum Dots/Poly(l-Glutamic Acid)–Drug Conjugates for Drug Delivery and Cell Imaging. Analyst.

[15] Korepanov O.A., Mazing D.S., Aleksandrova O.A., Moshnikov V.A. (2019). Synthesis and Study of Colloidal Nanocrystals Based on Ternary Chalcogenides for Active Media of Heavy Metal Ions Sensors. Proceedings of the 2019 IEEE Conference of Russian Young Researchers in Electrical and Electronic Engineering (EIConRus).

[16] Jain S., Bharti S., Bhullar G.K., Tripathi S.K. (2022). Synthesis, Characterization and Stability Study of Aqueous MPA Capped CuInS_2_/ZnS Core/Shell Nanoparticles. J. Lumin..

[17] Mazing D.S., Chernaguzov I.S., Shulga A.I., Korepanov O.A., Aleksandrova O.A., Moshnikov V.A. (2018). Synthesis of Ternary Chalcogenide Colloidal Nanocrystals in Aqueous Medium. J. Phys. Conf. Ser..

[18] Liu Z., Ma Q., Wang X., Lin Z., Zhang H., Liu L., Su X. (2014). A Novel Fluorescent Nanosensor for Detection of Heparin and Heparinase Based on CuInS_2_ Quantum Dots. Biosens. Bioelectron..

[19] An X., Zhang Y., Wang J., Kong D., He X., Chen L., Zhang Y. (2021). The Preparation of CuInS_2_-ZnS-Glutathione Quantum Dots and Their Application on the Sensitive Determination of Cytochrome *c* and Imaging of HeLa Cells. ACS Omega.

[20] Amaral-Júnior J.C., Mansur A.A.P., Carvalho I.C., Mansur H.S. (2021). Tunable Luminescence of Cu-In-S/ZnS Quantum Dots-Polysaccharide Nanohybrids by Environmentally Friendly Synthesis for Potential Solar Energy Photoconversion Applications. Appl. Surf. Sci..

[21] Korepanov O., Aleksandrova O., Firsov D., Kalazhokov Z., Kirilenko D., Kozodaev D., Matveev V., Mazing D., Moshnikov V. (2022). Polyvinylpyrrolidone as a Stabilizer in Synthesis of AgInS_2_ Quantum Dots. Nanomaterials.

[22] Haouari M., Maaoui A., Saad N., Bulou A. (2017). Optical Temperature Sensing Using Green Emissions of Er^3+^ Doped Fluoro-Tellurite Glass. Sens. Actuators A Phys..

[23] Meert K.W., Morozov V.A., Abakumov A.M., Hadermann J., Poelman D., Smet P.F. (2014). Energy Transfer in Eu^3+^ Doped Scheelites: Use as Thermographic Phosphor. Opt. Express.

[24] Zhong J., Chen D., Peng Y., Lu Y., Chen X., Li X., Ji Z. (2018). A Review on Nanostructured Glass Ceramics for Promising Application in Optical Thermometry. J. Alloys Compd..

[25] Iida K., Kim D. (2022). Temperature-Dependent Photoluminescence Properties of Water-Soluble CuInS_2_ and CuInS_2_/ZnS Quantum Dots. J. Appl. Phys..

[26] Xia C., Wu W., Yu T., Xie X., Van Oversteeg C., Gerritsen H.C., De Mello Donega C. (2018). Size-Dependent Band-Gap and Molar Absorption Coefficients of Colloidal CuInS_2_ Quantum Dots. ACS Nano.

[27] Shi A., Wang X., Meng X., Liu X., Li H., Zhao J. (2012). Temperature-Dependent Photoluminescence of CuInS_2_ Quantum Dots. J. Lumin..

[28] Mir I.A., Das K., Akhter T., Ranjan R., Patel R., Bohidar H.B. (2018). Eco-Friendly Synthesis of CuInS_2_ and CuInS_2_@ZnS Quantum Dots and Their Effect on Enzyme Activity of Lysozyme. RSC Adv..

[29] Speranskaya E.S., Sevrin C., De Saeger S., Hens Z., Goryacheva I.Y., Grandfils C. (2016). Synthesis of Hydrophilic CuInS_2_/ZnS Quantum Dots with Different Polymeric Shells and Study of Their Cytotoxicity and Hemocompatibility. ACS Appl. Mater. Interfaces.

[30] Firsov D.D., Komkov O.S., Solov’ev V.A., Kop’ev P.S., Ivanov S.V. (2016). Temperature-Dependent Photoluminescence of InSb/InAs Nanostructures with InSb Thickness in the above-Monolayer Range. J. Phys. D Appl. Phys..

[31] Landry C.C., Barron A.R. (1993). Synthesis of Polycrystalline Chalcopyrite Semiconductors by Microwave Irradiation. Science.

[32] Chen B., Zhong H., Zhang W., Tan Z., Li Y., Yu C., Zhai T., Bando Y., Yang S., Zou B. (2012). Highly Emissive and Color-Tunable CuInS_2_-Based Colloidal Semiconductor Nanocrystals: Off-Stoichiometry Effects and Improved Electroluminescence Performance. Adv. Funct. Mater..

[33] Berends A.C., Rabouw F.T., Spoor F.C.M., Bladt E., Grozema F.C., Houtepen A.J., Siebbeles L.D.A., de Mello Donegá C. (2016). Radiative and Nonradiative Recombination in CuInS_2_ Nanocrystals and CuInS_2_-Based Core/Shell Nanocrystals. J. Phys. Chem. Lett..

[34] Fuhr A.S., Yun H.J., Makarov N.S., Li H., McDaniel H., Klimov V.I. (2017). Light Emission Mechanisms in CuInS_2_ Quantum Dots Evaluated by Spectral Electrochemistry. ACS Photonics.

[35] Zang H., Li H., Makarov N.S., Velizhanin K.A., Wu K., Park Y.-S., Klimov V.I. (2017). Thick-Shell CuInS_2_/ZnS Quantum Dots with Suppressed “Blinking” and Narrow Single-Particle Emission Line Widths. Nano Lett..

[36] Rinnert H., Jambois O., Vergnat M. (2009). Photoluminescence Properties of Size-Controlled Silicon Nanocrystals at Low Temperatures. J. Appl. Phys..

[37] Sun J., Ikezawa M., Wang X., Jing P., Li H., Zhao J., Masumoto Y. (2015). Photocarrier Recombination Dynamics in Ternary Chalcogenide CuInS_2_ Quantum Dots. Phys. Chem. Chem. Phys..

[38] Kalytchuk S., Zhovtiuk O., Kershaw S.V., Zbořil R., Rogach A.L. (2016). Temperature-Dependent Exciton and Trap-Related Photoluminescence of CdTe Quantum Dots Embedded in a NaCl Matrix: Implication in Thermometry. Small.

[39] Jing P., Zheng J., Ikezawa M., Liu X., Lv S., Kong X., Zhao J., Masumoto Y. (2009). Temperature-Dependent Photoluminescence of CdSe-Core CdS/CdZnS/ZnS-Multishell Quantum Dots. J. Phys. Chem. C.

[40] Joshi A., Narsingi K.Y., Manasreh M.O., Davis E.A., Weaver B.D. (2006). Temperature Dependence of the Band Gap of Colloidal CdSe/ZnS Core/Shell Nanocrystals Embedded into an Ultraviolet Curable Resin. Appl. Phys. Lett..

[41] Alphandéry E., Nicholas R.J., Mason N.J., Lyapin S.G., Klipstein P.C. (2002). Photoluminescence of Self-Assembled InSb Quantum Dots Grown on GaSb as a Function of Excitation Power, Temperature, and Magnetic Field. Phys. Rev. B.

[42] Eliseev P.G., Osinski M., Lee J., Sugahara T., Sakai S. (2000). Band-Tail Model and Temperature-Induced Blue-Shift in Photoluminescence Spectra of In_x_Ga_1-x_N Grown on Sapphire. J. Elec. Mater..

[43] Knowles K.E., Nelson H.D., Kilburn T.B., Gamelin D.R. (2015). Singlet–Triplet Splittings in the Luminescent Excited States of Colloidal Cu^+^:CdSe, Cu^+^:InP, and CuInS_2_ Nanocrystals: Charge-Transfer Configurations and Self-Trapped Excitons. J. Am. Chem. Soc..

[44] Bol A.A., Ferwerda J., Bergwerff J.A., Meijerink A. (2002). Luminescence of Nanocrystalline ZnS:Cu^2+^. J. Lumin..

[45] Suyver J.F., van der Beek T., Wuister S.F., Kelly J.J., Meijerink A. (2001). Luminescence of Nanocrystalline ZnSe:Cu. Appl. Phys. Lett..

[46] Stouwdam J.W., Janssen R.A.J. (2009). Electroluminescent Cu-Doped CdS Quantum Dots. Adv. Mater..

[47] Meulenberg R.W., van Buuren T., Hanif K.M., Willey T.M., Strouse G.F., Terminello L.J. (2004). Structure and Composition of Cu-Doped CdSe Nanocrystals Using Soft X-Ray Absorption Spectroscopy. Nano Lett..

[48] Uehara M., Watanabe K., Tajiri Y., Nakamura H., Maeda H. (2008). Synthesis of CuInS_2_ Fluorescent Nanocrystals and Enhancement of Fluorescence by Controlling Crystal Defect. J. Chem. Phys..

[49] Courtel F.M., Paynter R.W., Marsan B., Morin M. (2009). Synthesis, Characterization, and Growth Mechanism of n-Type CuInS_2_ Colloidal Particles. Chem. Mater..

[50] Miropoltsev M., Wegner K.D., Häusler I., Hodoroaba V.-D., Resch-Genger U. (2022). Influence of Hydrophilic Thiol Ligands of Varying Denticity on the Luminescence Properties and Colloidal Stability of Quaternary Semiconductor Nanocrystals. J. Phys. Chem. C.

[51] Karczewski G., Maćkowski S., Kutrowski M., Wojtowicz T., Kossut J. (1999). Photoluminescence Study of CdTe/ZnTe Self-Assembled Quantum Dots. Appl. Phys. Lett..

