# Supplementary material for: Temperature- and Size-Dependent Photoluminescence of CuInS2 Quantum Dots
Source: Nanomaterials (Basel). 2023 Nov 1;13(21):2892. doi: 10.3390/nano13212892 (PMC10650527; doi:10.3390/nano13212892)
Supplement: Supplementary file 1 [file nanomaterials-13-02892-s001.zip › nanomaterials-2671893-supplementary.pdf]

## Temperature- and Size-Dependent Photoluminescence of $CuInS_2$ Quantum Dots

According to TEM images (figure S1), the particles in fraction #1 are noticeably larger with sizes from around 4 nm, in fraction #5 the particles do not exceed 3 nm.

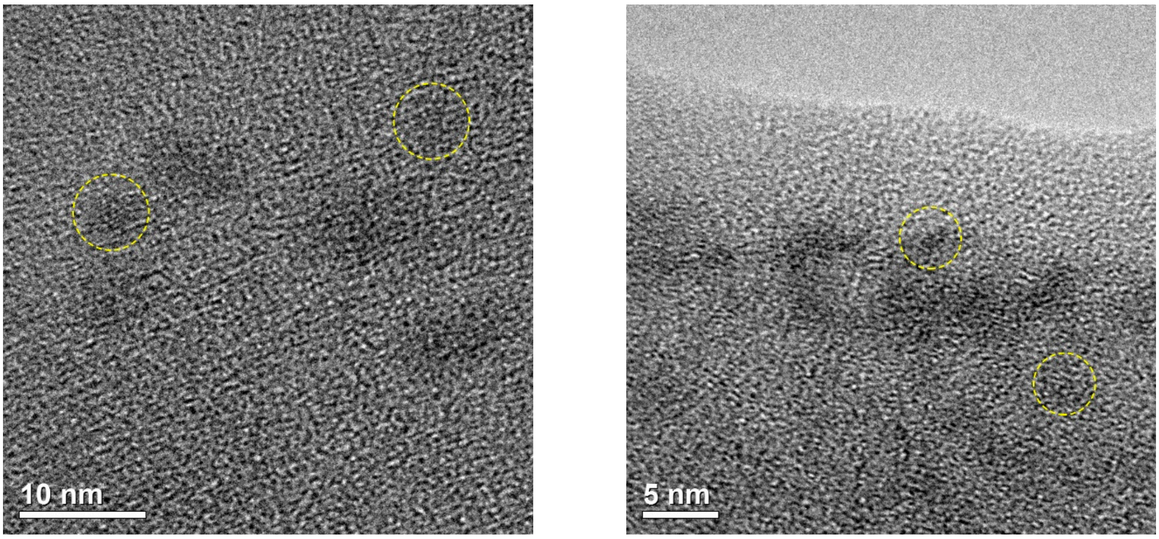


(**a**) (**b**)

**Figure S1.** TEM image of the fraction #1 (a) and fraction #5 (b) of $CuInS_2$ QDs

**Table S1.** PL parameters of size selected QDs in solution

| Fraction # | PL peak position, nm | FWHM, nm | FWHM, meV |
|---|---|---|---|
| 1 | 713 | 149 | 353 |
| 2 | 707 | 143 | 348 |
| 3 | 700 | 140 | 347 |
| 4 | 686 | 135 | 348 |
| 5 | 677 | 130 | 345 |

**Table S2.** $\Gamma_{inh}$ and $\Gamma_{LO}$ values calculated with the formula (4) for the different fractions

| Fraction # | $\Gamma_{inh}$, meV | $\Gamma_{LO}$, meV |
|---|---|---|
| 1 | 244 | 110 |
| 2 | 246 | 100 |
| 3 | 259 | 95 |
| 4 | 278 | 85 |
| 5 | 280 | 80 |

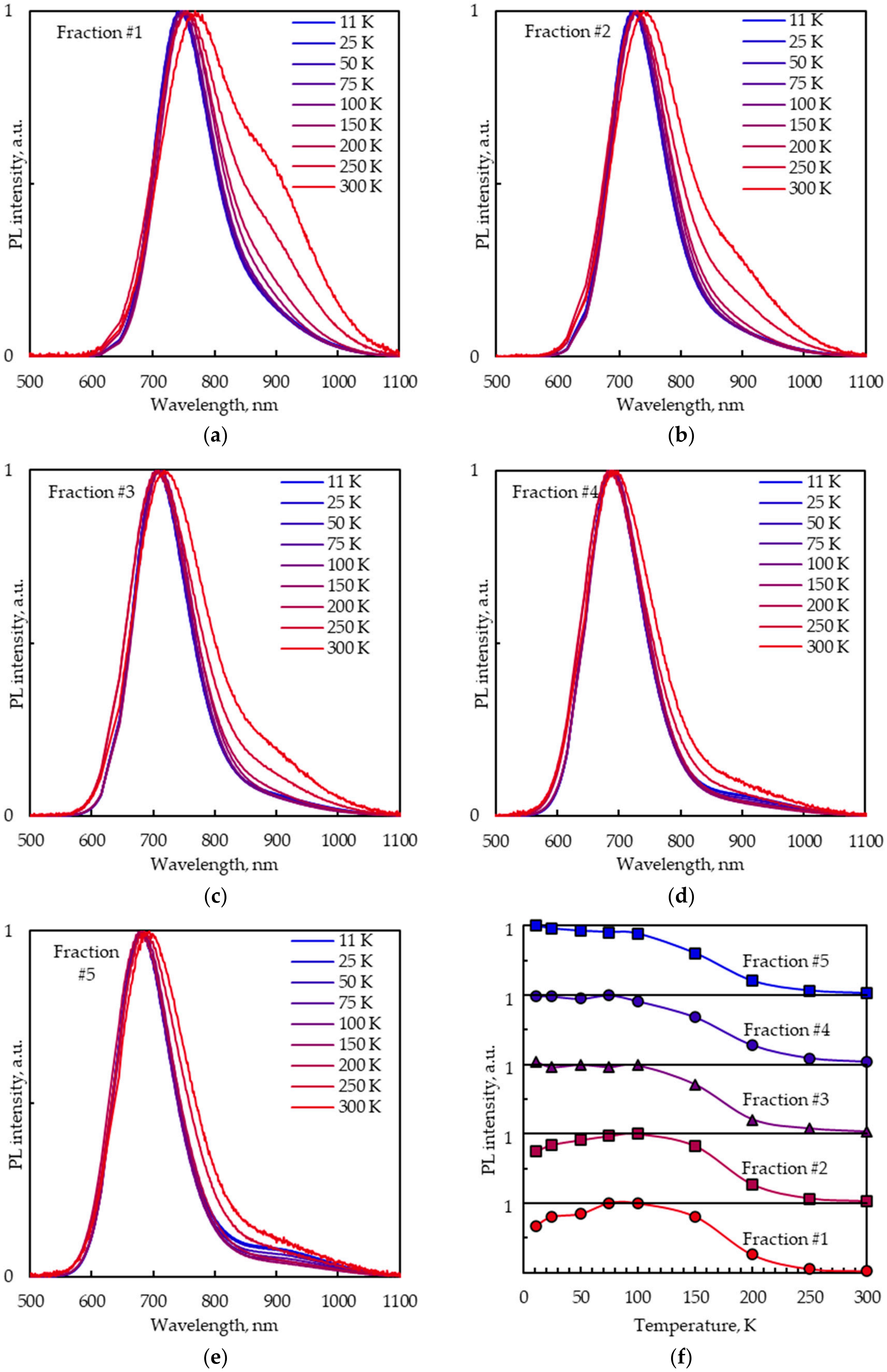


**Figure S2.** Normalized PL spectra of size-selected $CuInS_2$ QDs in KBr pellets in temperature range 11–300 K. Fractions #1–#5 presented in (**a**)–(**e**). Temperature dependence of the PL integral intensity of the same samples (**f**)
